# Study of the Molecular Recognition of Aptamers Selected through Ovarian Cancer Cell-SELEX

**DOI:** 10.1371/journal.pone.0013770

**Published:** 2010-11-01

**Authors:** Dimitri Van Simaeys, Dalia López-Colón, Kwame Sefah, Rebecca Sutphen, Elizabeth Jimenez, Weihong Tan

**Affiliations:** 1 Department of Chemistry, Center for Research at Bio/Nano Interface, University of Florida, Gainesville, Florida, United States of America; 2 Shands Cancer and Genetic Research Center, Department of Physiology and Functional Genomics, University of Florida, Gainesville, Florida, United States of America; 3 Department of Pediatrics, College of Medicine, University of South Florida, Tampa, Florida, United States of America; 4 Moffitt Cancer Center and Research Institute, Tampa, Florida, United States of America; University of Southampton, United Kingdom

## Abstract

**Background:**

Ovarian cancer is the most lethal gynecological malignancy, and the ovarian clear cell carcinoma subtype (OCCA) demonstrates a particularly poor response to standard treatment. Improvements in ovarian cancer outcomes, especially for OCCA, could be expected from a clearer understanding of the molecular pathology that might guide strategies for earlier diagnosis and more effective treatment.

**Methodology/Principal Findings:**

Cell-SELEX technology was employed to develop new molecular probes for ovarian cancer cell surface markers. A total of thirteen aptamers with K_d_'s to ovarian cancer cells in the pico- to nanomolar range were obtained. Preliminary investigation of the targets of these aptamers and their binding characteristics was also performed.

**Conclusions/Significance:**

We have selected a series of aptamers that bind to different types of ovarian cancer, but not cervical cancer. Though binding to other cancer cell lines was observed, these aptamers could lead to identification of biomarkers that are related to cancer.

## Introduction

Ovarian cancer is the fifth most common cancer in women [1], and has the highest death rate of any gynecologic malignancy. This disease is characterized by few early symptoms, presentation at an advanced stage in the majority of cases, and poor survival rates [2]–[8]. The prognosis is especially poor for patients with ovarian clear cell adenocarcinoma (OCCA), which is often resistant to standard platinum-based chemotherapy [6]–[10].

The most commonly used serum biomarker for clinical diagnosis and prognosis is ovarian cancer antigen 125 (CA-125). The CA-125 value is elevated in approximately 90% of late-stage cases of epithelial ovarian cancer (stages 3 and 4). However, it is only elevated in 50-60% of women with early stage disease and is also elevated in a number of benign conditions [2], [5], [7], [11]–[13].

The utilization of aptamers has great potential for the identification of new biomarkers. Aptamers, which are probes capable of specifically binding to cell surface markers expressed by targeted tumor cells [14]–[21], are short single-stranded oligonucleotides of about 100 nt. They are selected from large combinatorial pools of sequences by Systematic Evolution of Ligands by Exponential Enrichment (SELEX) for their capacity to bind to targets, which can range from small molecules to proteins or polysaccharides, as well as tumor cells [16]–[19], [21]–[23]. Aptamers have well-defined tertiary structures that dictate the selectivity for their targets.

The target specificity and affinity of aptamers are similar to those of antibodies, but with several advantages over antibodies for clinical use. Aptamers may be chemically synthesized in a short time at relatively low cost, allowing better batch-to-batch reproducibility and easier incorporation of chemical modifications. Since aptamers for cells are selected without prior knowledge of the target molecules, selected aptamers can be used to identify new surface markers on cancer cells [14], [16]–[18], [20], [24]–[27].

In this work, a total of 13 aptamers was selected for two model ovarian cancer cell lines: the OCCA line TOV-21G [28] (10 aptamers) and the ovarian serous adenocarcinoma line CAOV-3 [29] (3 aptamers). The cell surface targets of the aptamers were also briefly investigated. Preliminary investigation of the aptamers' targets and binding characteristics was also performed.

## Results and Discussion

Two model ovarian cancer cell lines were chosen for the selection of ovarian cancer aptamers: the OCCA cell line TOV-21G and the ovarian serous adenocarcinoma cell line CAOV-3. In order to identify aptamers that specifically bind to ovarian cancer cells, the cervical cancer cell line HeLa was used for counter-selection. The SELEX procedure for TOV-21G is described briefly below. A detailed description is provided in the experimental section.

**Figure 1:**
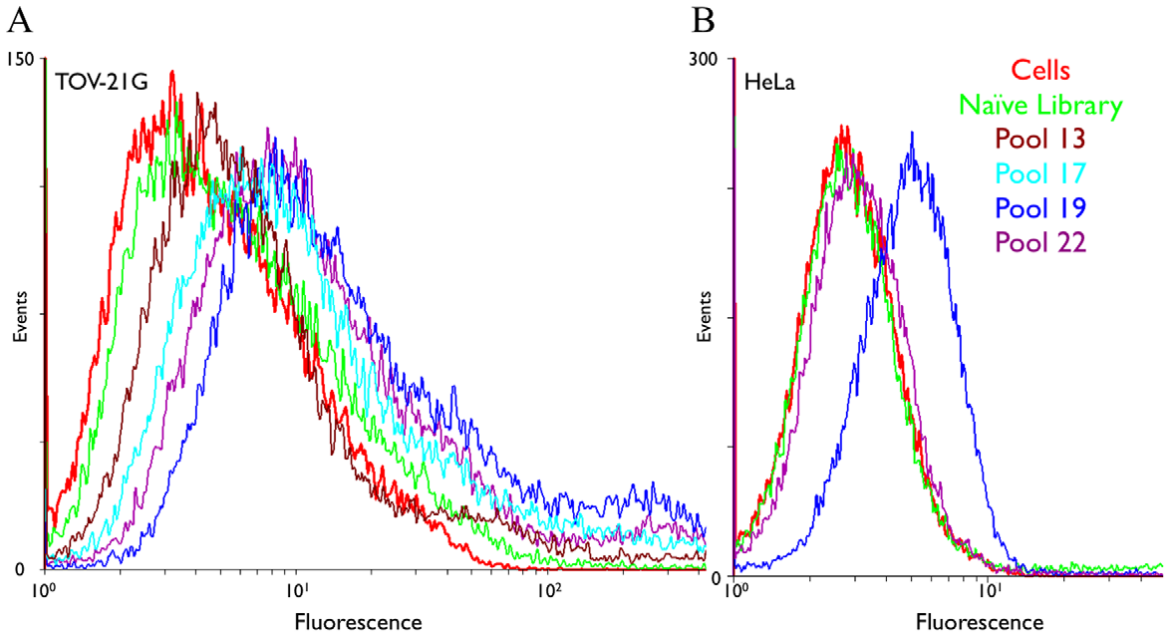
The binding assay of the enriched pools with TOV-21G and HeLa cells. A) The enrichment with TOV-21G cells B) The marginal binding of the respective pools to HeLa cells. By doing counter selection, sequences binding to HeLa were removed.

**Figure 2:**
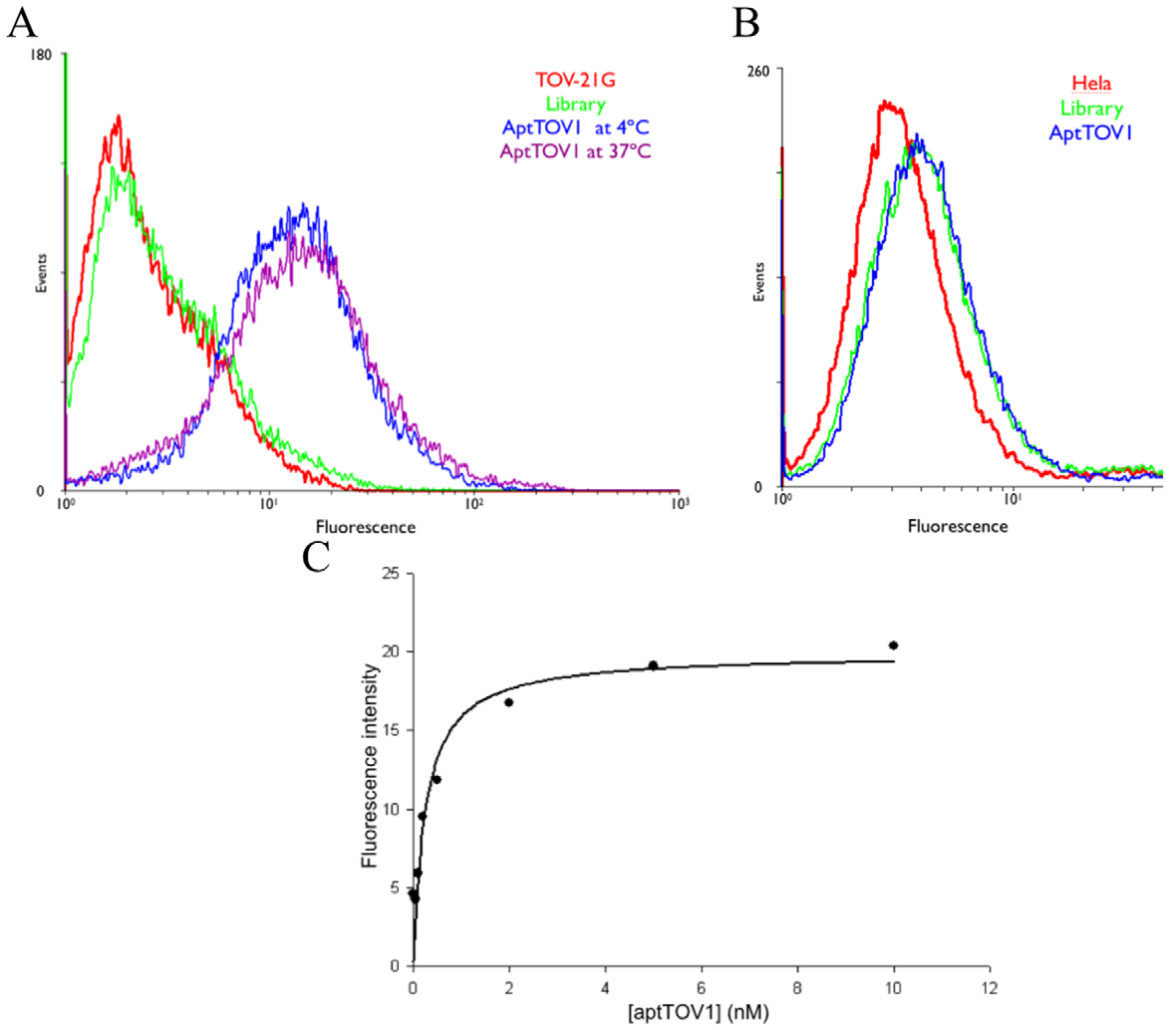
The binding of PE/cy5-labeled aptTOV1 (250 nM in binding buffer) A) to TOV-21G at 4°C and 37°C; B) to HeLa at 4°C. The negative control in these binding assays was PE/Cy5-labeled random library. C) Cells were incubated with varying concentrations of PE-Cy5-labeled aptamer in duplicate. The fluorescence intensity originating from background binding at each concentration was subtracted from the mean fluorescence intensity of the corresponding aptamer.

To start the selection process, 20 pmol of naive library was enriched by sequential binding to TOV-21G cell monolayers. Sequences showing non-specific binding to general cell surface markers were removed by incubating the enriched pool with HeLa cells (rounds 2, 4, 5, 7, 8, 9, 12, 20, 21, 22). The eluted pool for each round of SELEX was amplified through PCR, after which the ssDNA pools of interest were recovered and monitored for enrichment toward TOV-21G by flow cytometry. As the selected pools were enriched with sequences that recognize and bind to the target cell line, an increase in fluorescence signal was observed (Figure 1a). But attempts to omit counter-selection in rounds 13 to 19 led to enrichment for HeLa-binding sequences. The sequences binding to HeLa cells were successfully removed by counter selection in subsequent rounds, while the enrichment towards the target cell line was maintained (Figure 1b). After 22 rounds of SELEX, an enriched pool that specifically bound to the model OCCA cell line, but marginally to HeLa cells, was obtained (Figure 2). Thus, the pool was successfully enriched for sequences binding surface markers expressed by the model OCCA cell line, but not by cervical cancer cells.

Following completion of the selection process, three pools were chosen and submitted for sequencing: the final pool (round 22), the previous pool (round 21) and a pool showing minimal enrichment (round 13). Pool sequencing was used to help identify aptamer candidates by generating large quantities of sequences. This number of sequences (here a minimum of 2000 per pool) is large enough to allow identification of aptamers that only have a small representation in the pool (i.e. less then 1%). As can be seen in Table 1, selected aptamers were indeed present as early as a minimal enrichment was observed. AptTOV1's size percentage decreased as the SELEX continued, which is remarkable given the high affinity of this aptamer. This behavior has also been observed in other selections [30]. Other aptamers show a consistent increase in size percentage as the enrichment increased. The results from AptTOV6 are included to demonstrate that even relatively small families can lead to aptamers.

**Table 1:** The evolution of aptamers throughout the sequenced pools.

	Pool 13 (%)	Pool 21 (%)	Pool 22 (%)
**aptTOV1**	7,56	1,91	2,53
**aptTOV2**	6,43	10,84	18,62
**aptTOV2a**	2,96	4,92	7,65
**aptTOV2all**	9,39	15,76	26,27
**aptTOV3**	4,05	7,97	8,74
**aptTOV6**	0,3	0,92	0,58

Rows represent specific aptamers' percentage in the sequenced pools. AptTOVall is the sum of both AptTOV2 and AptTOV2a. AptTOV are aptamers from the SELEX for TOV-21G cells.

Sequences were aligned into families according to sequence homology. The number of homologues was compared across the different sequenced pools to validate their enrichment through the selection procedure using basic bio- informatics. Ten sequences showing the best homology throughout the pools were selected as aptamer candidates, synthesized and tested for binding to the model ovarian cancer cell lines. All the candidates showed binding to TOV-21G, with binding affinities in the pico- to nano-molar range (Table 2). This demonstrates the potential of next generation sequencing in SELEX to become a powerful and reproducible method for the development of aptamers.

**Table 2:** A compendium of the aptamers obtained by selection vs cancers TOV-21G (aptTOV) or CAOV-3 (DOV).

Name	Sequence	K_d_ (nM)	% in pool
aptTOV1	5′ - ATC CAG AGT GAC GCA GCA GAT CTG TGT AGG ATC GCA GTG TAG TGG ACA TTT GAT ACG ACT GGC TCG ACA CGG TGG CTT A - 3′	0.25±0.08	2,53
aptTOV2	5′ - ATC CAG AGT GAC GCA GCA TAA TCT CTA CAG GCG CAT GTA ATA TAA TGA AGC CCA TCC ACC TGG ACA CGG TGG CTT A- 3′	0.90±0.25	18,62
aptTOV2a	5′ - ATC CAG AGT GAC GCA GCA CAA TCT CTA CAG GCG CAT GTA ATA TAA TGG AGC CTA TCC ACG TCG ACA CGG TGG CTT A- 3′	11±3	7,65
aptTOV3	5′ - ATC CAG AGT GAC GCA GCA CTC ACT CTG ACC TTG GAT CGT CAC ATT ACA TGG GAT CAT CAG TCG ACA CGG TGG CTT A- 3′	30±9	8,74
aptTOV4	5′ - ATC CAG AGT GAC GCA GCA GGC ACT CTT CAC AAC ACG ACA TTT CAC TAC TCA CAA TCA CTC TCG ACA CGG TGG CTT A- 3′	20±5	0,52
aptTOV5	5′ - ATC CAG AGT GAC GCA GCA CAA CAT CCA CTC ATA ACT TCA ATA CAT ATC TGT CAC TCT TTC TCG ACA CGG TGG CTT A- 3′	4.5±1.2	0,82
aptTOV6	5′ - ATC CAG AGT GAC GCA GCA CGG CAC TCA CTC TTT GTT AAG TGG TCT GCT TCT TAA CCT TCA TCG ACA CGG TGG CTT A- 3′	29±7	0,58
aptTOV7	5′ - ATC CAG AGT GAC GCA GCA CCA ACT CGT ACA TCC TTC ACT TAA TCC GTC AAT CTA CCA CTC TCG ACA CGG TGG CTT A- 3′	6.6±2.3	0,19
aptTOV8	5′ - ATC CAG AGT GAC GCA GCA CCA GTC CAT CCC AAA ATC TGT CGT CAC ATA CCC TGC TGC GCC TCG ACA CGG TGG CTT A- 3′	17±3	0,76
aptTOV9	5′ - ATC CAG AGT GAC GCA GCA GCA ACA CAA ACC CAA CTT CTT ATC TTT TCG TTC ACT CTT CTC TCG ACA CGG TGG CTT A- 3′	26±10	0,06
DOV 3	5′ -ACT CAA CGA ACG CTG TGG ATG CAGAGG CTA GGATCT ATA GGT TCGGAC GTC GAT GAG GAC CAG GAG AGC A - 3′	132±32	ND
DOV 4	5′ - ACT CAA CGA ACG CTG TGG AGG GCA TCAGAT TAG GAT CTA TAG GTTCGG ACA TCG TGA GGA CCA GGA GAG CA - 3′	40±20	ND
DOV 6	5′ - ACT CAA CGA ACG CTG TGG AAT GTT GGGGTA GGT AGA AGG TGA AGGGGT TTC AGT TGA GGA CCA GGA GAG CA - 3′	39±20	ND

As shown in Table 2, aptamers aptTOV1 (K_d_  = 0.25±0.08 nM) and aptTOV2 (0.90±0.25 nM) bind very tightly to TOV-21G cells. As shown in Table 2, both aptamers can distinguish TOV-21G from HeLa cells.

The binding of the selected aptamers was tested with different adenocarcinoma cell lines, as well as other types of cancer cell lines, as shown in Table 2. Five of the aptamers selected against TOV-21G showed binding to CEM cells (acute lymphoblastic leukemia), while none of the aptamers bound to Ramos cells (Burkitt's lymphoma) or HL-60 (acute promyelocytic leukemia). All of the obtained aptamers bind to colorectal adenocarcinoma (HCT-116) and glioblastoma (A172). The aptamers obtained from TOV-21G do not bind to DLD-1 (Dukes' type C colorectal adenocarcinoma), while the aptamers coming from CAOV-3 did bind to this cell line. This behavior is similar to that observed in previous work conducted in our laboratory.

Since both selections took place at 4°C, the selected aptamers were tested at physiological conditions. The aptamers were incubated with the target cell line at 37°C and 4°C and their binding was measured. All aptamers showed similar binding at 4°C and 37°C (e.g., aptTOV1 in Figure 2a), suggesting that they have potential for in vivo studies.

**Figure 3:**
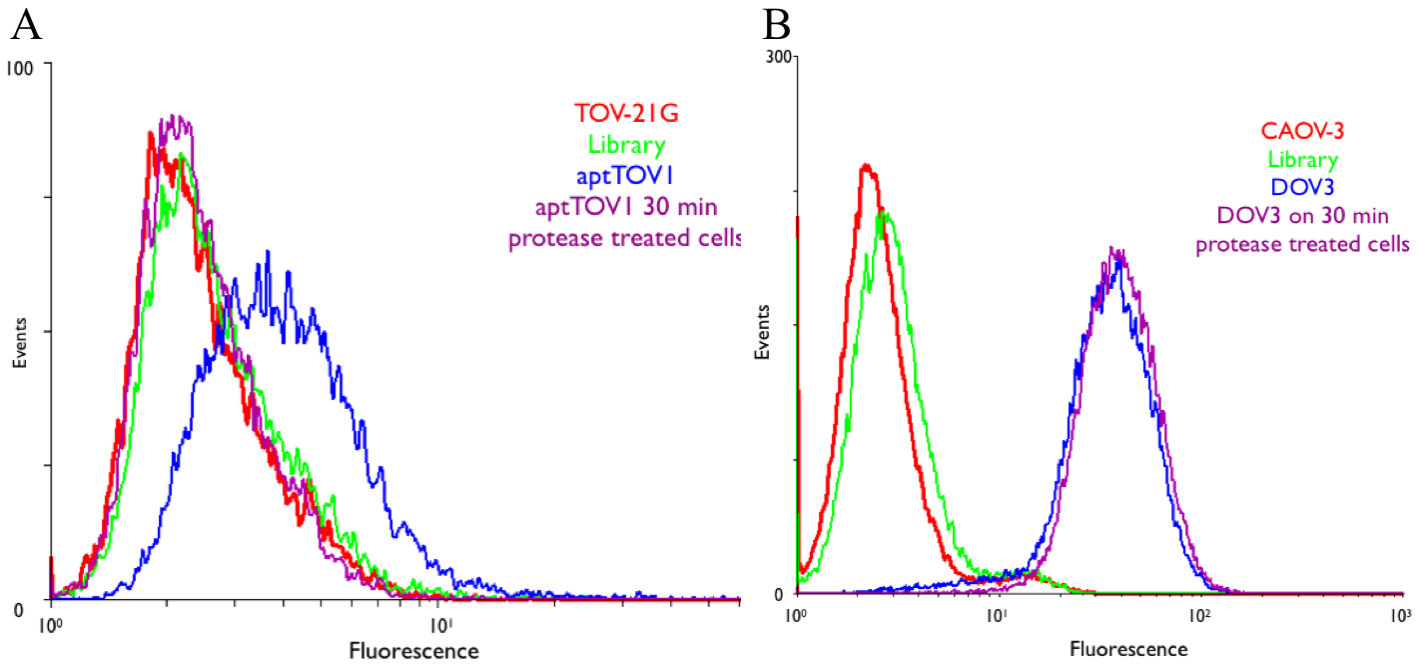
A preliminary study of the nature of the targets. A) No binding was observed for aptTOV1 to trypsinized TOV-21G cells. B) The binding of DOV 3 and 4 to CAOV-3 cells was not affected by protease treatment. All other aptamers showed the same behavior as represented in a).

To investigate the nature of the target molecule of each aptamer, binding of each aptamer was tested after treatment of the target cells with the proteases trypsin or proteinase K. As can be seen in Figure 3a, aptTOV1 shows a clear loss of binding to TOV-21G after protease treatment. The same behavior was also observed with all other aptTOV aptamers. Interestingly, for the second model cell line, CAOV-3, DOV3 and DOV4 retained their binding after protease treatment (Figure 3b), suggesting that these aptamers may not be binding to cell surface membrane proteins, but rather to another type of cell surface marker (i.e., carbohydrate or lipid). Neither DOV3 nor DOV4 binds to the tested leukemia cells (Table 3).

**Table 3:** Relative binding of the selected aptamers to various cell lines.

	TOV- 21G	CAOV-3	HeLa	BCC	H23	HT-29	HCT-116	A172	Ramos	CEM	HL- 60	DLD-1
**aptTOV1**	+++	-	-	++	++	-	++	+	-	-	-	-
**aptTOV2**	+++	-	-	++	-	-	++	++	-	-	-	-
**aptTOV2a**	+++	-	+	++	+	-	++	++	-	-	-	-
**aptTOV3**	++	-	-	++	+	-	++	++	-	-	-	-
**aptTOV4**	+++	+	-	++	++	-	++	+++	-	+	-	-
**aptTOV5**	+++	+	+	+++	++	+	++	+++	-	+++	-	-
**aptTOV6**	+++	-	-	++	++	-	++	+++	-	++	-	-
**aptTOV7**	+++	-	-	++	++	-	++	+++	-	++	-	-
**aptTOV8**	+++	-	-	+++	+	-	++	+++	-	+	-	-
**aptTOV9**	+++	-	-	++	+	-	++	++	-	-	-	-
**DOV3**	++++	++++	+++	+++	+++	+++	++++	++++	-	-	-	+++
**DOV4**	++++	++++	+++	++++	+++	+++	++++	++++	-	-	-	+++
**DOV6**	-	++	+	-	-	-	+	+	-	-	-	++

A dash indicates no observed binding to the corresponding cell line. See supplemental data S4 for our guidelines for the amount of pluses.

In conclusion, we have selected a series of aptamers with high affinity for ovarian cancer cells, including OCCA (TOV-21G) and serous adenocarcinoma (CAOV-3). By counter selection against HeLa cells, aptamers that can distinguish ovarian cancer from cervical cancer were selected. In particular, AptTOV 1 showed very high affinity towards TOV-21G, with a K_d_ of 250 pM.

Given the limited number of biomarkers for ovarian cancer currently available, the aptamers obtained from these selections have potential for improving diagnosis and treatment of this deadly disease. Because the aptamers also bind benign cysts (Table 3), the aptamers cannot be used to identify ovarian cancer *per se*. However, since the aptTOV apamers do not bind to a cancer of similar etiology (CAOV3) and also not to HeLa, they still have the potential to provide more insight into the pathology of ovarian cancer. It has been observed that there are significant differences in the proteome of serous and clear cell ovarian cancer [31]. The targets for these aptamers are most likely down regulated or silenced in these two cell models. Additionally, the AptTOV aptamers show binding to cancer cell lines from different non-related cancers (Table 3), and some AptTOV aptamers also bind CEM cells. This result suggests that the aptamers obtained from this SELEX can be used for profiling the expression of membrane proteins of different cancers. Identifying the targets of the selected aptamers is expected to shed light on the underlying mechanisms involved.

The discovery of two aptamers that were insensitive to protease digestion is intriguing. Additionally, their binding to all tested adenocarcinoma cells, but not to any of the leukemia cell lines, suggests the potential to further elucidate the underlying molecular differences between these cancer types. Further investigation is warranted to identify the targets of these aptamers and assess their performance in clinical samples.

## Materials and Methods

### Instrumentation and reagents

All oligonucleotides were synthesized by standard phosphoramidite chemistry using a 3400 DNA synthesizer (Applied Biosystems) and were purified by reversed-phase HPLC (Varian Prostar). All PCR mixtures contained 50 mM KCl, 10 mM Tris-HCl (pH 8.3), 2.0 mM MgCl_2_, dNTPs (each at 2.5 mM), 0.5 µM of each primer, and Hot start Taq DNA polymerase (5units/µL). PCR was performed on a Biorad Thermocycler and all reagents were purchased from Takara. Monitoring of pool enrichment, characterization of the selected aptamers, and identification of the target protein assays were performed by flow cytometric analysis using a FACScan cytometer (BD Immunocytometry Systems). Trypsin and Proteinase K were purchased from Fisher Biotech. The imaging of cells was performed with an Olympus FV500-IX81 confocal microscope (Olympus America Inc., Melville, NY). The DNA sequences were determined by the Genome Sequencing Services Laboratory at the University of Florida with the use of 454 sequencing (Roche).

### Cell culture and buffers

The CAOV-3, HeLa, Hs832(C)T and TOV-21G cell lines where obtained from the American Type Cell Culture (ATCC). The CAOV-3 and TOV-21G ovarian cancer cell lines where maintained in culture with MCBD 105: Medium 199 (1∶1); the HeLa cell line was cultured in RPMI-1640; and the Hs832(C)T cell line was cultured in Dulbecco's Modified Eagle's Medium (DMEM). All media where supplemented with 10% FBS and 100 UI/mL Penicillin-Streptomycin. Other cell lines used for selectivity assays included CEM (T cell leukemia), Ramos (Burkitt's Lymphoma), HCT-116, DLD-1, HT-29 (colorectal adenocarcinoma), NCI_H23 (non-small cell lung Cancer) and A172 (glioblastoma), all of which were cultured according to ATCC specifications. All cell lines where incubated at 37°C in a 5% CO_2_ atmosphere.

During the selection, cells were washed before and after incubation with wash buffer (WB), containing 4.5 g/L glucose and 5 mM MgCl_2_ in Dulbecco's phosphate buffered saline with calcium chloride and magnesium chloride (Sigma). Binding buffer (BB) used for selection was prepared by adding yeast tRNA (0.1 mg/mL) (Sigma) and BSA (1 mg/mL) (Fisher) to the wash buffer to reduce background binding.

### SELEX library and primers

The HPLC-purified library contained a segment of randomized sequence of 40 nucleotides (nt) flanked by 20-nt primer hybridization sites:

(5′- ATC CAG AGT GAC GCA GCA (N)_40_ TGG ACA CGG TGG CTT AGT-3′) and (5′-ACT ACC AAC GAG CGA CCA CT (N)_40_ AGA GTT CAG GAG AGG CAG GT-3′). The forward primers were labeled with 5′-FITC and the reverse primers were labeled with 5′-biotin.

### In Vitro cell-SELEX

In this study, TOV-21G was used as the target cell line and HeLa was used for counter-selection. For the first round, the cells were incubated with 20 pmol of naïve ssDNA library dissolved in BB. For later rounds, 50 pmol of enriched pool were used for incubation, also dissolved in BB. Before incubation, the ssDNA pool was denatured by heating at 95°C for 5 min and was cooled rapidly on ice for 5 min, allowing each sequence to form the most stable secondary structure.

The cells were washed twice (2 min) with WB and incubated with the DNA pool on ice in an orbital shaker for 30 min. In later selection rounds, the cells were washed with increased stringency to remove weakly binding sequences (a larger number of washes and increased washing time, up to 5 min). The bound sequences were eluted in 500 µL BB by heating at 95°C for 15 min, cooled on ice for 5 min and centrifuged at 14,000 rpm for 2 min.

The supernatant containing the DNA sequences was then incubated with a negative cell line to perform a subtraction of general sequences, as described above. The remaining sequences were amplified by PCR using the FITC- and biotin-labeled primers. Amplifications were carried out at 95°C for 30 s, 60°C for 30 s, and 72°C for 30 s, followed by final extension for 3 min at 72°C. The selected sense ssDNA was separated from the biotinylated antisense ssDNA by streptavidin-coated sepharose beads (Amersham Bioscience). The ssDNA was eluted from the sepharose beads by melting in a 0.2M NaOH solution.

The enrichment of specific sequences was assayed using flow cytometry as explained below. When the level of enrichment reached a plateau, pools of interest were submitted for sequencing. The aptamer selection for the CAOV3 cell line was performed using the same protocol, however a 1 minute trypsinization step was used to suspend the cells before adding the pools. Supplemental data S1 and S2 contain the clustal data of the alignments of each selection (final pool).

### Affinity studies: Flow cytofluorometric analysis for the determination of binding affinity

To determine the binding affinities of the aptamers, the target cells (5×10^5^) were incubated with various concentrations of 5′-biotin labeled aptamers on ice for 20 min in 100 µL of BB. Cells were then washed twice with 500 µL of BB, and suspended in 100 µL of BB containing streptavidin-PE-Cy5.5. Cells were then washed twice with 500 µL of WB, and were suspended in 200 µL of BB for flow cytometric analysis, using a 5′-biotin labeled random sequence as the negative control. All the experiments for binding assays were repeated at least 2 times. The specific binding intensity was calculated by subtraction of the mean fluorescence intensity of the background binding from the mean fluorescence intensity of the aptamers. The equilibrium dissociation constant (K_d_) of the fluorescent ligand was obtained by fitting a plot of the specific binding intensity versus (Y) the aptamer concentration (X) to the equation Y = B_max_X/(K_d_+X) using SigmaPlot. (Jandel, San Rafael, CA). Supplemental data S3 contains the flow data for all experiments presented in this article.

### Selectivity and specificity

To determine the cell specificity of the selected aptamers, cell lines including HeLa, K562, H23, H69, A172, HL-60. HT-29, Ramos and CEM were used in binding assays by flow cytometry as described above.

### Effect of temperature on aptamer binding

The aptamer selection process and all of the binding assays were performed on ice. It has been observed that some of the aptamers selected at lower temperatures may not bind well at 37°C [26], leading to poor performance under physiological conditions. In order to verify binding stability, aptamers were incubated with the target at 37°C, and fluorescence intensity was determined by flow cytometry. Aptamers incubated on ice were used as the positive control.

### Protease digestion assay

Target cells (5×10^5^) were detached using non-enzymatic cell dissociation solution. After resuspension, the cells were washed with 3 mL of PBS and then incubated with 1 mL of 0.05% trypsin/0.53 mM EDTA in HBSS or 0.1 mg/mL proteinase K in PBS at 37°C for 1, 5, 15, 30 and 60 minutes. Pure FBS was added to quench the proteinases. After washing with 2 mL of BB, the treated cells were used for binding assays as described above.

## Supporting Information

Data S1 Allignment of the final TOV pool.
(1.13 MB TXT)

Data S2 Allignment of the final CAOV3 pool.
(0.20 MB TXT)

Data S3 This file contains the flow data from the figures presented in this article.
(3.69 MB ZIP)

Data S4 Legend to the amount of pluses. This figure was our guideline to determine the amount of pluses for table 3.
(0.04 MB PDF)

## References

[1] Jemal A, Siegel R, Ward E, Hao Y, Xu J (2008). Cancer statistics, 2008.. CA Cancer J Clin.

[2] Hartge P (2010). Designing early detection programs for ovarian cancer.. J Natl Cancer Inst.

[3] Kurman RJ, Shih IM (2010). The Origin and Pathogenesis of Epithelial Ovarian Cancer: A Proposed Unifying Theory.. Am J Surg Pathol.

[4] Willmott LJ, Fruehauf JP (2010). Targeted therapy in ovarian cancer.. J Oncol.

[5] Nosov V, Su F, Amneus M, Birrer M, Robins T (2009). Validation of serum biomarkers for detection of early-stage ovarian cancer.. Am J Obstet Gynecol.

[6] Crotzer DR, Sun CC, Coleman RL, Wolf JK, Levenback CF (2007). Lack of effective systemic therapy for recurrent clear cell carcinoma of the ovary.. Gynecol Oncol.

[7] Munkarah A, Chatterjee M, Tainsky MA (2007). Update on ovarian cancer screening.. Curr Opin Obstet Gynecol.

[8] Reynolds EA, Moller KA (2006). A review and an update on the screening of epithelial ovarian cancer.. Curr Probl Cancer.

[9] Shirai T, Imanaka Y, Sekimoto M, Ishizaki T (2009). Primary chemotherapy patterns for ovarian cancer treatment in Japan.. J Obstet Gynaecol Res.

[10] Williams TI, Toups KL, Saggese DA, Kalli KR, Cliby WA (2007). Epithelial ovarian cancer: disease etiology, treatment, detection, and investigational gene, metabolite, and protein biomarkers.. J Proteome Res.

[11] Maines-Bandiera S, Woo MM, Borugian M, Molday LL, Hii T (2010). Oviductal glycoprotein (OVGP1, MUC9): a differentiation-based mucin present in serum of women with ovarian cancer.. Int J Gynecol Cancer.

[12] Oaknin A, Barretina P, Perez X, Jimenez L, Velasco M (2010). CA-125 response patterns in patients with recurrent ovarian cancer treated with pegylated liposomal doxorubicin (PLD).. Int J Gynecol Cancer.

[13] Jacobs IJ, Menon U (2004). Progress and challenges in screening for early detection of ovarian cancer.. Mol Cell Proteomics.

[14] Fang X, Tan W (2010). Aptamers generated from cell-SELEX for molecular medicine: a chemical biology approach.. Acc Chem Res.

[15] Lu C, Shahzad MM, Moreno-Smith M, Lin YG, Jennings NB (2010). Targeting pericytes with a PDGF-B aptamer in human ovarian carcinoma models.. Cancer Biol Ther.

[16] Ulrich H, Wrenger C (2009). Disease-specific biomarker discovery by aptamers.. Cytometry A.

[17] Zhao Z, Xu L, Shi X, Tan W, Fang X (2009). Recognition of subtype non-small cell lung cancer by DNA aptamers selected from living cells.. Analyst.

[18] Chen HW, Medley CD, Sefah K, Shangguan D, Tang Z (2008). Molecular recognition of small-cell lung cancer cells using aptamers.. ChemMedChem.

[19] Shangguan D, Cao Z, Meng L, Mallikaratchy P, Sefah K (2008). Cell-specific aptamer probes for membrane protein elucidation in cancer cells.. J Proteome Res.

[20] Shangguan D, Cao ZC, Li Y, Tan W (2007). Aptamers Evolved from Cultured Cancer Cells Reveal Molecular Differences of Cancer Cells in Patient Samples.. Clin Chem.

[21] Cerchia L, Duconge F, Pestourie C, Boulay J, Aissouni Y (2005). Neutralizing aptamers from whole-cell SELEX inhibit the RET receptor tyrosine kinase.. PLoS Biol.

[22] Bing T, Yang X, Mei H, Cao Z, Shangguan D (2010). Conservative secondary structure motif of streptavidin-binding aptamers generated by different laboratories.. Bioorg Med Chem.

[23] Jhaveri S, Rajendran M, Ellington AD (2000). In vitro selection of signaling aptamers.. Nat Biotechnol.

[24] Tang Z, Parekh P, Turner P, Moyer RW, Tan W (2009). Generating aptamers for recognition of virus-infected cells.. Clin Chem.

[25] Shangguan D, Meng L, Cao ZC, Xiao Z, Fang X (2008). Identification of liver cancer-specific aptamers using whole live cells.. Anal Chem.

[26] Mallikaratchy P, Tang Z, Kwame S, Meng L, Shangguan D (2007). Aptamer directly evolved from live cells recognizes membrane bound immunoglobin heavy mu chain in Burkitt's lymphoma cells.. Mol Cell Proteomics.

[27] Shangguan D, Li Y, Tang Z, Cao ZC, Chen HW (2006). Aptamers evolved from live cells as effective molecular probes for cancer study.. Proc Natl Acad Sci U S A.

[28] Provencher DM, Lounis H, Champoux L, Tetrault M, Manderson EN (2000). Characterization of four novel epithelial ovarian cancer cell lines.. In Vitro Cell Dev Biol Anim.

[29] Bast RC, Jacobs I, Berchuck A (1992). Malignant transformation of ovarian epithelium.. J Natl Cancer Inst.

[30] Fitter S, James R (2005). Deconvolution of a Complex Target Using DNA Aptamers.. JBC.

[31] Faca V M, Ventura A P, Fitzgibbon M P, Pereira-Faca S R et al (2008). Proteomic Analysis of ovarian cancer cells reveals dynamic processes of protein secretion and shedding of extra-cellular domains.. PLoS ONE.

