# Supplementary material for: Study of the Molecular Recognition of Aptamers Selected through Ovarian Cancer Cell-SELEX
Source: PLoS One. 2010 Nov 1;5(11):e13770. doi: 10.1371/journal.pone.0013770 (PMC2967474; doi:10.1371/journal.pone.0013770)
Supplement: Data S4 — Legend to the amount of pluses. This figure was our guideline to determine the amount of pluses for table 3. (0.04 MB PDF) [file pone.0013770.s004.pdf]

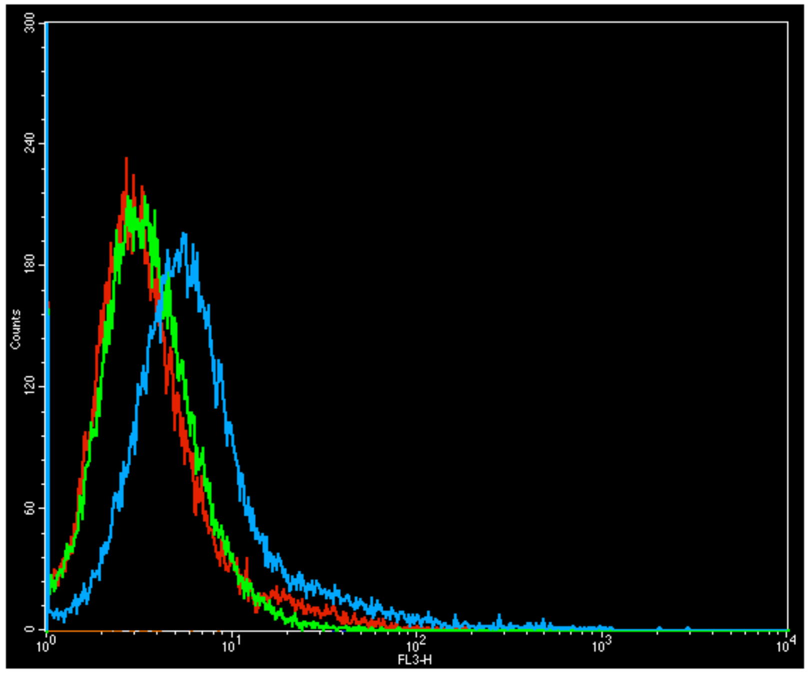

+10-30%

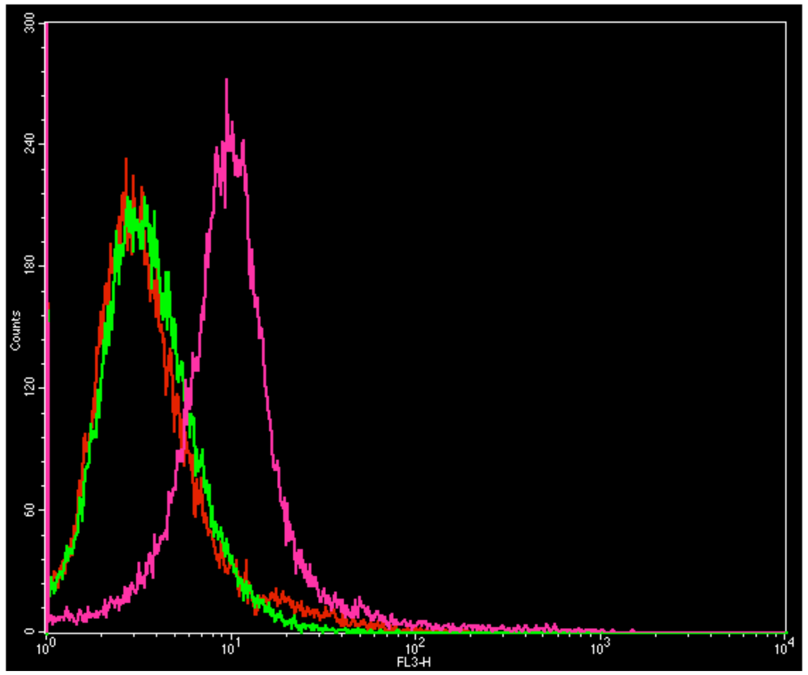

++ 30-60%

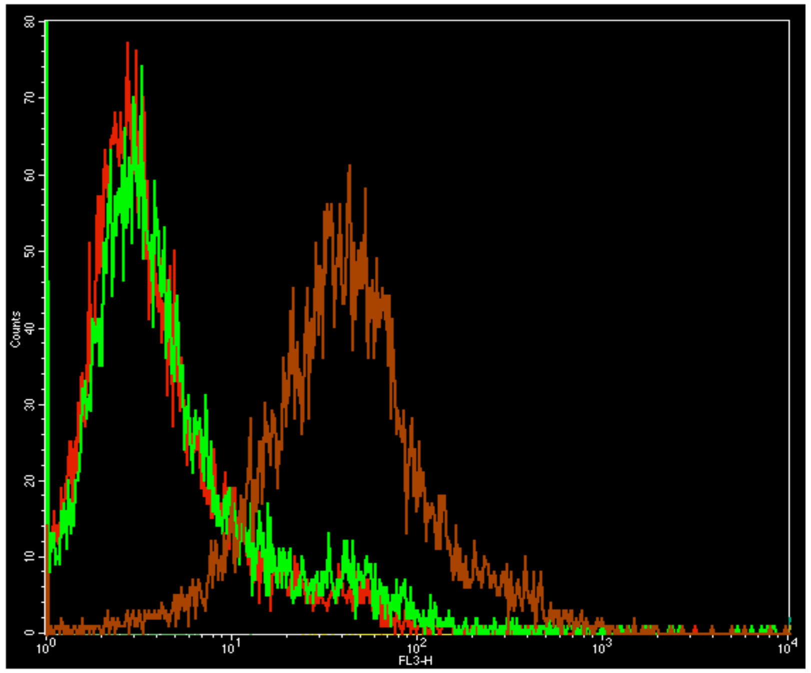

+++ 60-80%

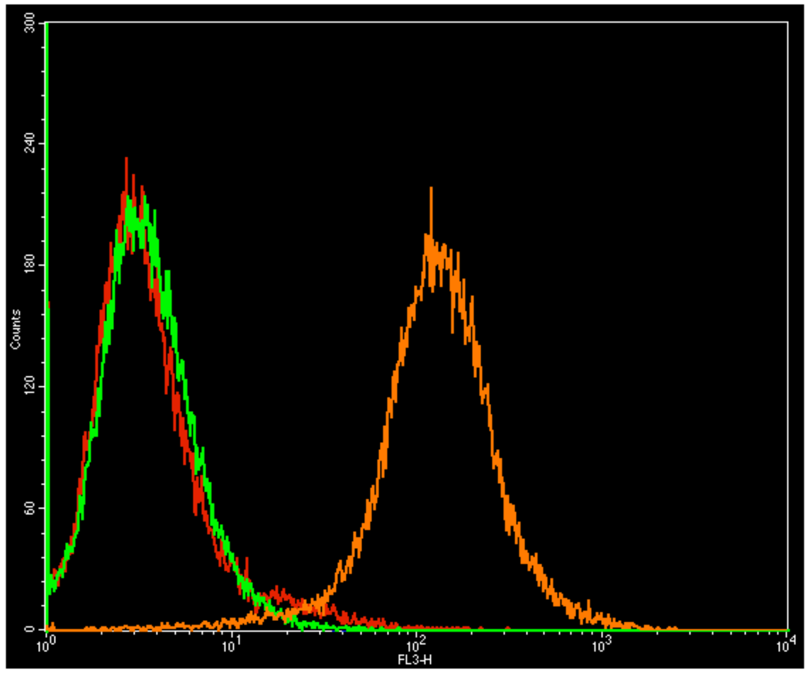

++++ >80%

A threshold based on the fluorescence intensity of the PE-Cy5.5-labeled library was chosen so that 99% of the cells have fluorescence intensities below it. When PE-cy5.5 labeled aptamer was incubated with the cells, the percentage of cells with fluorescence intensity above the set threshold was used to evaluate the binding
